# Prevention of atherosclerosis by Yindan Xinnaotong capsule combined with swimming in rats

**DOI:** 10.1186/s12906-015-0622-7

**Published:** 2015-04-08

**Authors:** Jianlu Wang, Lan Wang, Hongjun Yang, Yun You, Haiyu Xu, Leilei Gong, Xiaojie Yin, Wandan Wang, Shuangrong Gao, Long Cheng, Rixin Liang, Fulong Liao

**Affiliations:** China Academy of Chinese Medical Sciences, Institute of Chinese Material Medical, Beijing, China; School of Traditional Chinese Medicine, Capital Medical University, Beijing, China; Chinese Academy of Medical Sciences, Institute of Medicinal Plant Development, Beijing, China; Guizhou Bailing Group Pharmaceutical Co., Ltd, Guizhou, China

**Keywords:** Atherosclerosis, Swimming, Shear stress, Yindan Xinnaotong capsule, Lipid-lowering, Endothelial protection

## Abstract

**Background:**

Yindan Xinnaotong capsule has been used for treating cardio-cerebrovascular diseases for several decades in China. Exercise training can protect against the development of atherosclerosis. The aim of the present study is to evaluate the joint effect of YXC and exercise on atherosclerosis in rats.

**Methods:**

A combined method involving low shear stress and a high-fat diet was used to establish the atherosclerosis model in rats. Partial ligation of the left common carotid artery was performed, and then the rats were divided into 9 treatment groups according to a 3 × 3 factorial design with two factors and three levels for each factor, swimming of 0, 0.5, 1 h daily and YXC administration of 0, 1, 2 g/kg p.o. daily. Next the interventions of swimming and YXC were executed for 8 weeks. After that, blood samples were collected to determine blood viscosity, plasma viscosity, haematocrit (HCT), fibrinogen (FIB), blood lipid profile (including total cholesterol (TC), low-density lipoprotein-cholesterol (LDL-C), triglyceride (TG) and high-density lipoprotein-cholesterol (HDL-C)), nitric oxide (NO), 6-keto- prostaglandin (PG) F1α, endothelin (ET) and thromboxane (TX) B_2_. The common carotid arteries of the rats were harvested to examine pathological changes, wall thickness and circumference, and the expression of SM22αwas assayed via immune-histochemistry.

**Results:**

The early pathological changes were observed. The joint effects of YXC and swimming showed significant changes in the examined parameters: (1) decreases in plasma viscosity, blood viscosity and FIB; (2) increases in NO and 6-keto-PGF1α; (3) decreases in ET and TXB_2_; and (4) decreases in LDL-C and TG. The combination of 2 g/kg YXC and 1 h of swimming led to synergistic decreases in LDL-C and TG. The interactive effect between YXC and swimming was obvious in decreasing wall thickness. Swimming alone was able to up-regulate the expression of SM22α.

**Conclusions:**

In conclusion, this study indicates that the combination of YXC and swimming may prevent atherosclerosis through a synergistic effect between YXC and swimming in improving blood circulation, hemorheological parameters, blood lipids levels and the vascular endothelium in rats. The vascular remodeling may be contributed to the prevention effects on AS by up-regulating SM22α.

## Background

Atherosclerosis (AS) is a pathological condition that underlies several important adverse vascular events such as angina, myocardial infarction, strock, which are recognized as leading causes of morbidity and mortality worldwide. The World Health Organization (WHO) predicts that heart diseases and stroke are becoming more deadly, a projected combined death toll will reach 24 million by 2030 [1].

AS is a complex and multifactorial process. Although the pathogenic role of lipid retention and inflammation in AS is widely aware, shear stress–related events should deserve more concerns. Caro and his co-workers [2,3] showed that hemodynamic factors have been thought to be highly relevant to AS [4-6]. Recent studies have found that low shear stress (<4 dynes/cm2) might be involved in the development of AS [7], but high shear stress contributes to an anti-atherosclerotic effect [8].

Influence of turbulent blood flow on lesion development of AS is closely associated with endothelium. Eendothelial cells sense wall shear stress and transfer this information to stimulate secretion of inflammatory cytokine. If this inflammatory cytokine still exist, it will result in proliferation and migration of vascular smooth muscle cells and further remodeling of vascular [9-11]. A large body of evidences have demonstrated that shear stress within the physiological range elicit NO release in cultured endothelial cells [12]. Grabowski showed that shear stress could increase the levels of PGI2 in endothelial cells [13].

In addition, other events such as endothelial dysfunction, inflammation, lipoprotein oxidation, retention and aggregation [14-16] have been implicated in AS. Endothelial dysfunction is considered as a first initiator in AS, which is characterized by the decrease of nitric oxide (NO), the increase of endothelin (ET) and alteration in the production of prostanoids, which can provoke atherosclerotic lesion formation [17-19]. Inflammation is also necessary and sufficient to promote initiation and development of AS. Some inflammatory makers including C-reactive protein (CRP), interleukin-1β (IL-1β) and interleukin-18 (IL-18) are regarded as the indicators of AS development [20,21].

There are also many investigators considering subendothelial retention of atherogenic lipoproteins as the key pathogenic process in atherogenesis. Several lines of evidence indicate that hyperlipidemia can lead to lesion development in specific sites in the existence of predisposing stimuli, such as sheer stress. Following lipoprotein retention caused by abundant atherogenic lipoproteins, aggregation promptly occurs or may be part of the retentive process. Once significant retention has occurred, the early responses of AS, including lipoprotein oxidation and cellular chemotaxis, accelerate AS lesion development [16].

Ross et al. had also proposed that atherosclerosis is progressive disease characterized by the accumulation of lipids and fibrous elements in the arterial walls and is the primary cause of heart disease and stroke. In the earliest stages of atherosclerosis, cholesterol accumulates in several locations and bring about the formation of foam cells, lead to growth of atherosclerotic lesion [22].

Some researchers agree that exercise can also help to prevent AS, in addition to medication and dietary control [23]. Okabe Taka-aki [24] and Shimada et al. [25] showed that swimming could suppress the development of AS plaques in the carotid artery in apoE deficient mice, and this effect occurs via the antioxidant action of the nitric oxide system. Other scholars concluded that physical exercise can affect local vascular and systemic inflammation in AS through decreasing inflammation and endothelial dysfunction [26]. Biomechanopharmacology by Fulong Liao [27] believes that the joint effects of medication and biomechanical factors (including flow shear stress) play an important role in preventing AS. Yun You et al. [28] demonstrated that the combination of an oral SL (SL, the Chinese abbreviation for Radix Salviae miltiorrhizae and Andrographis paniculata) extract with swimming inhibited inflammatory factors, improved hemorheological parameters and lipid profile in rat model of AS.

Traditional Chinese medicine (TCM) with the efficacy of activating blood circulation and eliminating stasis function is beneficial for cardio-and cerebrovascular diseases in China [29]. Yindan Xinnaotong capsule (YXC) is composed of *Ginkgo biloba, Radix Salviae miltiorrhizae, Gynostemma pentaphyllum, Erigeron breviscapus, Allium.sativam L.var.Viviparum Regel, Panax notoginseng, Crataegus pinnatifida Bge.* and *Borneolum syntheticum* (the ratio of each medicine was 50:50:30:30:40:20:40:1), and indicates an anti-atherosclerotic effect in animal experiments and clinical application, and the mechanism may involve decreasing blood lipid concentrations, anti-inflammatory or anti-coagulation properties, protecting the vascular endothelium and improving microcirculation [30-33]. However, the effects of YXC on vascular remodeling have been poorly described in the literature.

Exercise can protect against the development of AS via regulating blood flow shear stress, and YXC showed anti-atherosclerosis action, so this study was designed to test the hypotheses that there may be some synergistic or addictive effects between exercise and YXC in preventing atherosclerosis. For the attempt, a factorial design of two factors, swimming and YXC, was employed.

## Methods

### Animal preparation and surgery protocol

Eighty male Sprague–Dawley rats (180–200 g) were purchased from the experimental animal centre of the Chinese People’s Liberation Army (PLA) Military Academy of Medical Science (SCXK2007-004; Beijing, China). They were divided into nine interventional groups (n =8, each) and one sham-operated group (n = 8). All animal experiments were approved by the Laboratory Animal Ethics Committee of the Institute of Basic Theory of TCM, China Academy of Chinese Medical Sciences (Beijing, China). All animals were handled in accordance with international ethics requirements. The approve code of Ethics Committee was SYXK (Jing) 2005–2008 and the period of validity was from Aug, 2011 to Aug, 2015.

An animal model of early AS in rats was established by ligation of the left common carotid artery (LCCA) to induce vascular stenosis. Stenosis of the LCCA was established by the method described by Fang Hua [34], Shi Fang Ding [35] and Douglas Nam [36] with some modifications. Briefly, after the rats were anaesthetised using 10% chloral hydrate at 0.3 g/kg via intraperitoneal injection, the surgical area was epilated and disinfected with betadine. Then, a surgical incision was made at the midline of the neck while the rats were in a supine position, and the LCCA and external carotid artery bifurcation were carefully isolated, without damage to the vessels or recurrent laryngeal nerve. A segment of suture (5–0) was placed around the LCCA, and a sterile acupuncture needle with an outer diameter of 0.30 mm (Wuxi Jiajian Medical Instrument Company, Wuxi China) was placed under the suture parallel to the LCCA. The needle was tied tightly together with the LCCA using the suture at a distance of 1.5 cm from the bifurcation. The needle was then quickly removed, and the tied suture remained around the LCCA. Thus, the inner diameter of the stenosed portion of the LCCA was about 0.3 mm, and a LCCA stenosis was formed. Finally, a single intramuscular injection of gentamicin sulphate (3.5 ml/kg) was administered after the incision was closed. The sham-operated group underwent the same process without ligation of the artery.

The blood flow in the LCCA was monitored continuously before and after surgery using a multichannel electrophysiolograph, and the degree of stenosis was evaluated. The stenosis rate was 46 ± 8%. After the carotid surgery, the rats were placed in a cage and held at 28°C for 3 h, then returned to the animal care room and fed a normal diet. Thereafter, 6 × 10^5^ IU · kg-1 vitamin D_3_ was intraperitoneally injected once per day for 2 days, except for the sham-operated group.

### YXC and exercise protocol

One week after surgery, the rats in the sham-operated group were fed a normal diet, whereas the rats in the other groups were fed a high-fat diet (1% cholesterol, 0.2% pig bile salts, 10% lard, 10% egg yolk powder, 78.8% basal diet, which was purchased from Beijing Keao Xieli Feed Co., LTD, China). The intervention described below was applied for 8 weeks in all groups, except the sham-operated group.

A factorial design involving two factors (swimming and YXC) with three levels was adopted. The two factors were swimming and YXC (Guizhou Bailing Group Pharmaceutical Co., LTD, China. Lot number: 20120214). The three levels of each factor were as follows: swimming (no swimming, 0.5 hours per day, 1 hour per day) and YXC capsule (no YXC, 1 g/kg per day, 2 g/kg per day). Thus, nine groups were included in this 3 × 3 factorial design.

A dose of 1 g/kg YXC in rats is approximately equal to 4.8 g per day for a human weighing 60 kg. So, 1 g/kg YXC is so-called clinical equivalent dose in pharmacology. The rats were orally administered a mixed liquid consisting of YXC dissolved in purified water once daily for 6 days per week.

The swimming duration was selected according to Yun You [28] and Taka-aki Okabe [24]. The rats were placed passively in a plastic swimming pool for the exercise training (100 cm × 60 cm × 80 cm) at 35 ± 1°C. The exercise duration was gradually increased with a step of 10 min on each subsequent day, starting from 10 min and extending up to 30 min or 60 min, 5 days per week. The whole swimming procession was supervised to ensure that every rat underwent the exercise training during the period of swimming.

### Hemorheological parameters and physicochemical properties

All rats were fasted in the night of the last day of the 8th week, and anaesthesia of 10% chloral hydrate at a dose of 0.3 g/kg was administered via intraperitoneal injection on the first day of the 9th week. Blood samples were collected through the abdominal aorta. During this process, three steps were carried out. The first 3 ml of blood was collected using 3.8% sodium citrate (1:9) as an anti-coagulant for hemorheological measurements, including determination of blood viscosity, hematocrit (HCT), plasma viscosity, fibrinogen (FIB) and thrombin time (TT). The second, 2 ml sample was collected using 7.5% disodium ethylene diamine tetraacetic acid (EDTA) and aprotinin as anti-coagulants and spun at 3,000 rpm for 15 min to determine the contents of ET, 6-keto-PGF1α and thromboxane (TX) B_2_ in the serum. The final, 3 ml whole-blood sample, without anti-coagulants, was centrifuged at 3,000 rpm for 15 min, and the serum was retained for determination of total cholesterol (TC), triglyceride (TG), low-density lipoprotein-cholesterol (LDL-C), high-density lipoprotein-cholesterol (HDL-C) and NO.

Blood viscosity under shear rates of 10 s^−1^ and 200 s^−1^ at 37°C was tested with a blood viscometer (LBY-N6A, Precil Co.). Plasma viscosity at 37°C was tested with a capillary viscometer (LBY-NW1, Precil Co.). HCT was determined via the capillary centrifugation method. FIB (Shanghai Sunbio Co., LTD., lot: 136042) and TT (Shanghai Sunbio Co., LTD., lot: 121082) were determined using test kits.

Blood lipid profile was measured with an automatic biochemistry analyser according to the manufacturer’s protocol (GFD-800; Gaomi Caihong Analyser Co., Ltd., Shan-dong China), including TC, TG, LDL-C and HDL-C. The four testing kits were all purchased from Beijing BHKT Clinical Reagents Co., Ltd., China, with lot numbers of 20120115, 20130112, 20130401 and 20130507 accordingly.

The plasma concentrations of ET, 6-keto-PGF1α and TXB_2_ were determined via radioimmunoassay using commercially available kits. The three kits were obtained from the Beijing Northern Bio-technology Research Institute, with a same lot number of 20120620. The serum concentration of NO was measured using a commercially available kit according to the manufacturer’s instructions (Nanjing Jiancheng Bioengineering Institute, lot: 20130624).

### Evaluation of the vascular remodeling of the carotid artery

After blood samples were collected from the abdominal aorta, sections of the LCCA of 2 cm from the ligation on either side were harvested, then fixed with 4% paraformaldehyde and paraffin-embedded. Serial sections were taken right and left separately near ligation. Carotid artery sections (4 μm) were stained with hematoxylin and eosin (HE), the images were obtained at × 200 magnification and histopathological evaluations were performed using 3–5 sections from different locations of the carotid artery.

Vascular remodeling, including both structural and functional remodeling, was evaluated. Pathological changes were observed under an optical microscope (BX51, Olympus Company, Tokyo, Japan), and images were captured with an Olympus DP72 camera (Olympus Company, Tokyo, Japan). The wall thickness (namely from the innermost of intima to the outmost of media, and the direction of measurement was perpendicular to vessel wall) and vessel circumference were measured using DP2-BSW image-analysis software (Olympus Company, Tokyo, Japan). Three cross-sections from each aortic section were used for the analysis of wall thickness and vessel circumference, and the values were averaged.

Immunohistochemical analyses of SM22α expression were performed. The primary antibody for these analyses was the rabbit anti-rat SM22 alpha polyclonal antibody (dilution 1:200; Abcam Ltd., Hong Kong), which was used to distinguish the smooth muscle cells within the lesions. The secondary antibody was streptavidin marked by horse reddish peroxidise (Boster Bio co., LTD, Wuhan, China). Immunoreactivity was detected with the DAB Kit (Boster Bio co., LTD, Wuhan, China), and the product was identified by a brown colour. Phosphate buffer (PBS) replaced the SM22 alpha antibody in the negative control, and the other steps remained the same.

Following immunohistochemistry staining, an Olympus imaging analysis system was employed to observe the vascular walls and light microscope slices in each of 3–5 randomly selected high-power fields. The integrated optical density (IOD) value of positive areas was measured with Image Pro Plus 6.0 imaging analysis software. In our analysis, we used the relative level of positive expression, defined as the ratio of positive expression IOD to the negative control IOD.

### Statistical analysis

Statistical analysis was performed with SPSS version 11.5 (SPSS, Chicago, IL, USA). All data are expressed as the mean ± SD. The GLM procedure was applied for a two-way ANOVA to test for synergism. Comparisons between each pair of factor levels were analyzed using a Bonferon post hoc test. A value of *P* < 0.05 was considered statistically significant.

## Results

### Hemorheological measurements

**Figure 1:**
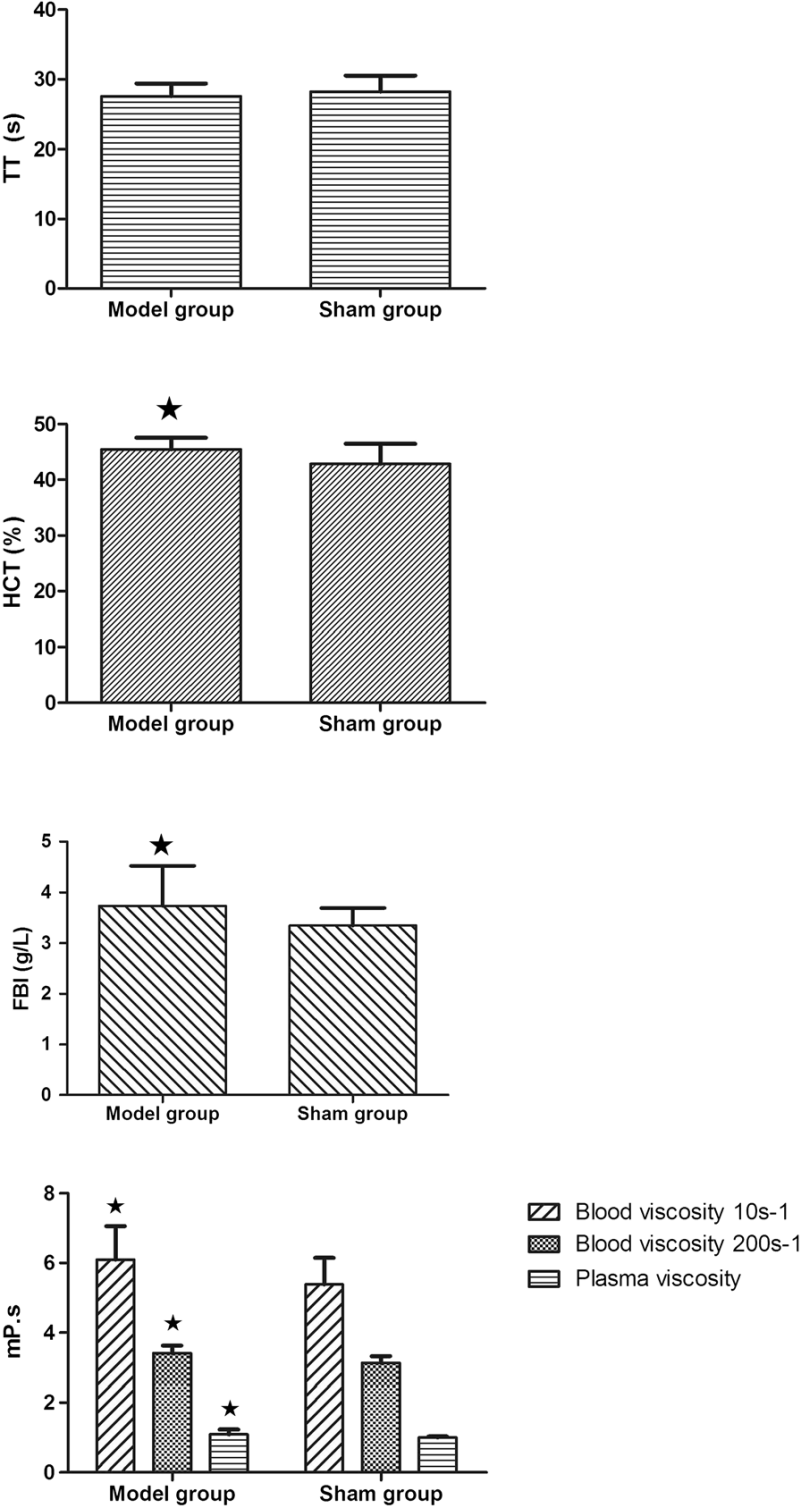
**Bar graphs of hemorheological parameters compared between model group and sham-operated group.** All data are expressed as the mean ± SD. Comparisons between sham group and model group of factor levels were analyzed using Bonferon. A value of *P* < 0.05 was considered statistically significant.

The values of plasma viscosity, blood viscosity at shear rates of 10 s^−1^ and 200 s^−1^, FIB and HCT in the model group were significantly higher than those in the sham-operated rats and TT was lower (see Figure 1).

There were significant interactive effects between YXC and swimming in decreasing plasma viscosity, blood viscosities, FIB and HCT, suggesting YXC and swimming could jointly improve hemorheological properties markedly and the combination of the two factors could have a synergistic effect (see Table 1).

**Table 1:** **Hemorheological parameters of different treatment (mean ± SD) (n = 8, each)**

**YXC (g/kg)**	**Swimming (h)**	**Plasma viscosity(mP.s)**		**Blood viscosity (mP.s)(10s** ^**−1**^ **)**		**Blood iscosity (mP.s)(200 s** ^**-1**^ **)**		**FIB (g/L)**		**HCT (%)**		**TT (s)**	
0	0	1.09 ± 0.14		6.10 ± 0.96		3.41 ± 0.22		3.74 ± 0.78		45.46 ± 2.10		27.57 ± 1.83	
0	0.5	1.00 ± 0.05		4.92 ± 0.77		3.02 ± 0.20		4.94 ± 1.20		41.23 ± 4.68		28.59 ± 2.24	
0	1	0.99 ± 0.04		6.09 ± 0.73		3.20 ± 0.09		3.57 ± 0.67		45.44 ± 1.61		27.20 ± 0.99	
1	0	1.03 ± 0.07		5.24 ± 0.97		3.05 ± 0.30		4.14 ± 1.03		41.35 ± 5.71		27.47 ± 0.85	
1	0.5	1.04 ± 0.05		5.50 ± 0.60		3.12 ± 0.21		3.81 ± 0.60		42.32 ± 3.75		27.94 ± 1.67	
1	1	1.01 ± 0.02		5.27 ± 0.89		3.05 ± 0.22		3.58 ± 0.20		41.24 ± 4.12		28.45 ± 1.77	
2	0	0.98 ± 0.02		5.08 ± 0.87		2.95 ± 0.21		3.77 ± 0.29		40.66 ± 2.95		27.28 ± 1.41	
2	0.5	1.00 ± 0.04		5.64 ± 0.67		3.18 ± 0.13		3.44 ± 0.27		44.03 ± 2.48		27.37 ± 1.09	
2	1	1.01 ± 0.05		5.45 ± 0.84		3.18 ± 0.24		3.70 ± 0.74		42.55 ± 4.51		28.17 ± 1.01	
Two-way ANOVA		*F*	*P*	*F*	*P*	*F*	*P*	*F*	*P*	*F*	*P*	*F*	*P*
YXC		2.365	0.101	1.496	0.237	3.153	0.049	2.413	0.098	2.720	0.073	0.168	0.845
Swimming		1.707	0.177	0.601	0.551	0.236	0.791	2.065	0.135	0.205	0.815	0.831	0.440
YXC × Swimming		3.276	0.016	3.192	0.018	5.263	0.001	4.052	0.005	2.554	0.047	0.855	0.495

All data are expressed as the mean ± SD. The GLM procedure was applied for a two-way ANOVA to test for synergism effect. Comparisons between each pair of factor levels were analyzed using a Bonferon post hoc test. A value of *P* < 0.05 was considered statistically significant.

### Blood lipoprotein measurements

**Figure 2:**
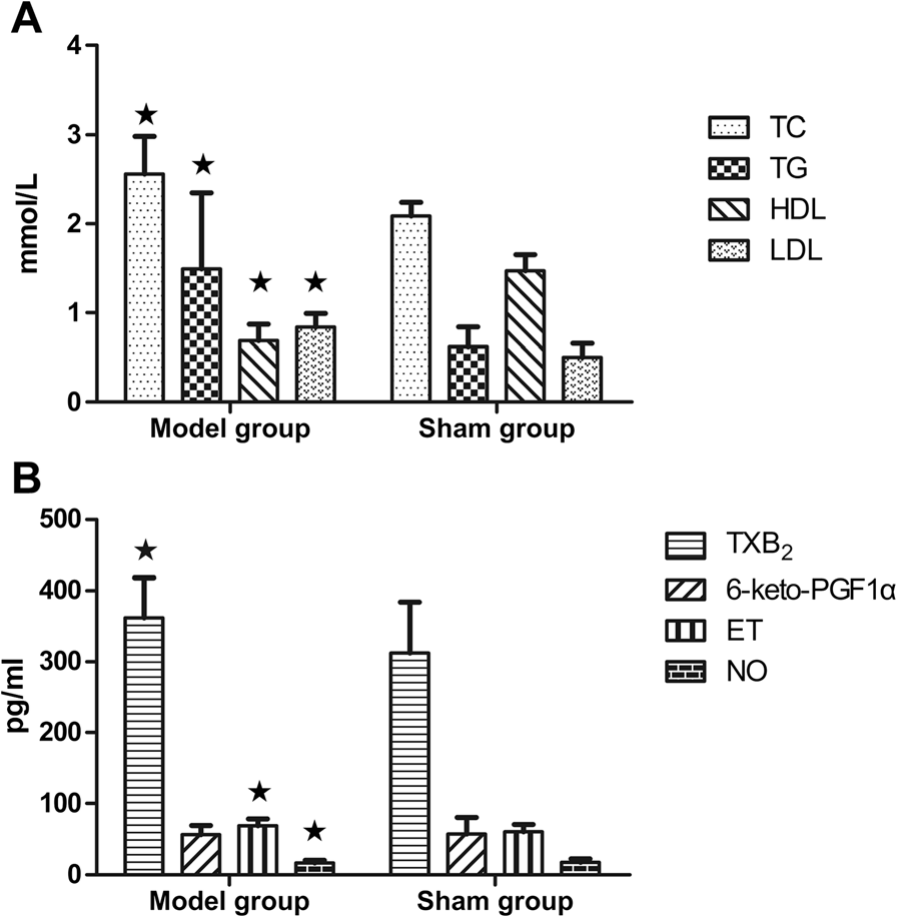
**Bar graphs of serum lipoproteins and endothelial factors compared between model group and sham-operated group. A)** Comparison of serum lipoproteins between model group and sham-operated group; **B)** Comparison of endothelial factors between model group and sham-operated group. All data are expressed as the mean ± SD. Comparisons between sham group and model group of factor levels were analyzed using Bonferon. A value of *P* < 0.05 was considered statistically significant.

Compared to the sham-operated group, the levels of serum TC, LDL-C and TG in the model group were significantly higher (p < 0.05), while the levels of HDL-C were significantly lower (p < 0.05) (see Figure 2A).

The significant interactive effects between YXC and swimming on LDL-C and TG were found, indicating that the combination of YXC and swimming could lead to a marked decrease in blood lipoprotein level, while no significant interaction effects between YXC and swimming on TC and HDL-C were observed (see Table 2). The effect of swimming on LDL-C was obvious, suggesting that exercise training is helpful for the decrease of LDL-C instead of YXC. Treatment with 2 g/kg YXC exerted a decrease in TC and an increase in HDL-C (see Table 2).

**Table 2:** **Blood lipid profile of different treatment (mean ± SD) (n = 8, each)**

**YXC (g/kg)**	**Swimming (h)**	**TC (mmol/L)**		**LDL (mmol/L)**		**TG (mmol/L)**		**HDL (mmol/L)**	
0	0	2.56 ± 0.42		0.84 ± 0.15		1.49 ± 0.86		0.69 ± 0.18	
0	0.5	2.39 ± 0.40		1.00 ± 0.33		0.95 ± 0.50		0.94 ± 0.52	
0	1	2.39 ± 0.45		0.82 ± 0.22		0.45 ± 0.07		0.84 ± 0.16	
1	0	2.10 ± 0.30		0.60 ± 0.23		0.62 ± 0.20		1.32 ± 0.30	
1	0.5	2.65 ± 0.18		0.92 ± 0.16		0.78 ± 0.38		1.27 ± 0.31	
1	1	2.38 ± 0.72		0.96 ± 0.28		1.24 ± 0.47		1.25 ± 0.32	
2	0	2.07 ± 0.16		0.88 ± 0.04		0.76 ± 0.42		1.17 ± 0.19	
2	0.5	2.31 ± 0.55		0.94 ± 0.31		0.68 ± 0.28		1.12 ± 0.33	
2	1	1.77 ± 0.36		0.70 ± 0.10		0.47 ± 0.18		1.06 ± 0.20	
Two-way ANOVA		*F*	*P*	*F*	*P*	*F*	*P*	*F*	*P*
YXC		4.098	0.022	0.419	0.660	2.387	0.098	10.628	0.000
Swimming		1.929	0.155	3.459	0.038	1.396	0.249	0.219	0.804
YXC × Swimming		1.782	0.146	3.950	0.028	5.480	0.001	0.566	0.689

All data are expressed as the mean ± SD. The GLM procedure was applied for a two-way ANOVA to test for synergism effect. Comparisons between each pair of factor levels were analyzed using a Bonferon post hoc test. A value of *P* < 0.05 was considered statistically significant.

### Endothelial factor measurements

First of all, the levels of NO in the model rats decreased significantly (p < 0.05) and the levels of 6-keto-PGF1α were reduced, whereas the levels of ET and TXB_2_ were increased significantly (p < 0.05), compared to the rats in the sham-operated group (see Figure 2B).

There still existed significant interactive effects between YXC and swimming in increasing NO and 6-keto-PGF1α and decreasing ET and TXB2 (p < 0.05). YXC made a greater contribution to increasing NO (F = 37.361, p = 0.000). Swimming showed a main effect on decreasing TXB_2_ (F = 8.018, p = 0.001). The combination of 0.5 h swimming with 1 g/kg YXC led to the decrease of TXB_2_ with an increased 6-keto-PGF1α. The results proved that treatment of YXC and swimming jointly played a beneficial effect in improving endothelial functions (see Table 3).

**Table 3:** **Endothelial factors of different treatment (mean ± SD) (n = 8, each)**

**YXC (g/kg)**	**Swimming (h)**	**NO (pg/ml)**		**ET (pg/ml)**		**TXB**_**2**_ **(pg/ml)**		**6-keto-PGF**_**1α**_ **(pg/ml)**	
0	0	16.80 ± 3.99		69.54 ± 9.58		361.91 ± 56.38		57.08 ± 12.57	
0	0.5	22.67 ± 4.28		60.32 ± 9.99		207.85 ± 66.03		84.86 ± 32.33	
0	1	24.38 ± 4.47		66.04 ± 8.25		326.70 ± 155.64		78.76 ± 31.82	
1	0	40.38 ± 7.61		73.21 ± 5.25		531.44 ± 126.99		65.84 ± 25.92	
1	0.5	32.00 ± 9.56		60.21 ± 5.13		167.76 ± 86.16		63.27 ± 16.27	
1	1	33.07 ± 8.07		65.16 ± 9.22		345.63 ± 139.01		69.27 ± 17.32	
2	0	39.62 ± 6.61		53.55 ± 8.82		362.03 ± 76.92		105.91 ± 28.86	
2	0.5	42.29 ± 12.83		62.75 ± 6.91		385.41 ± 157.32		85.35 ± 16.71	
2	1	36.19 ± 6.51		64.14 ± 8.30		212.34 ± 90.98		45.26 ± 17.28	
**Two-way ANOVA**		***F***	***P***	***F***	***P***	***F***	***P***	***F***	***P***
YXC		37.361	0.000	3.642	0.330	0.778	0.466	1.544	0.224
Swimming		0.162	0.851	1.868	0.164	8.018	0.001	2.150	0.127
YXC × Swimming		3.142	0.020	4.418	0.004	5.479	0.001	6.322	0.001

All data are expressed as the mean ± SD. The GLM procedure was applied for a two-way ANOVA to test for synergism effect. Comparisons between each pair of factor levels were analyzed using a Bonferon post hoc test. A value of *P* < 0.05 was considered statistically significant.

### Histopathological assessment

**Figure 3:**
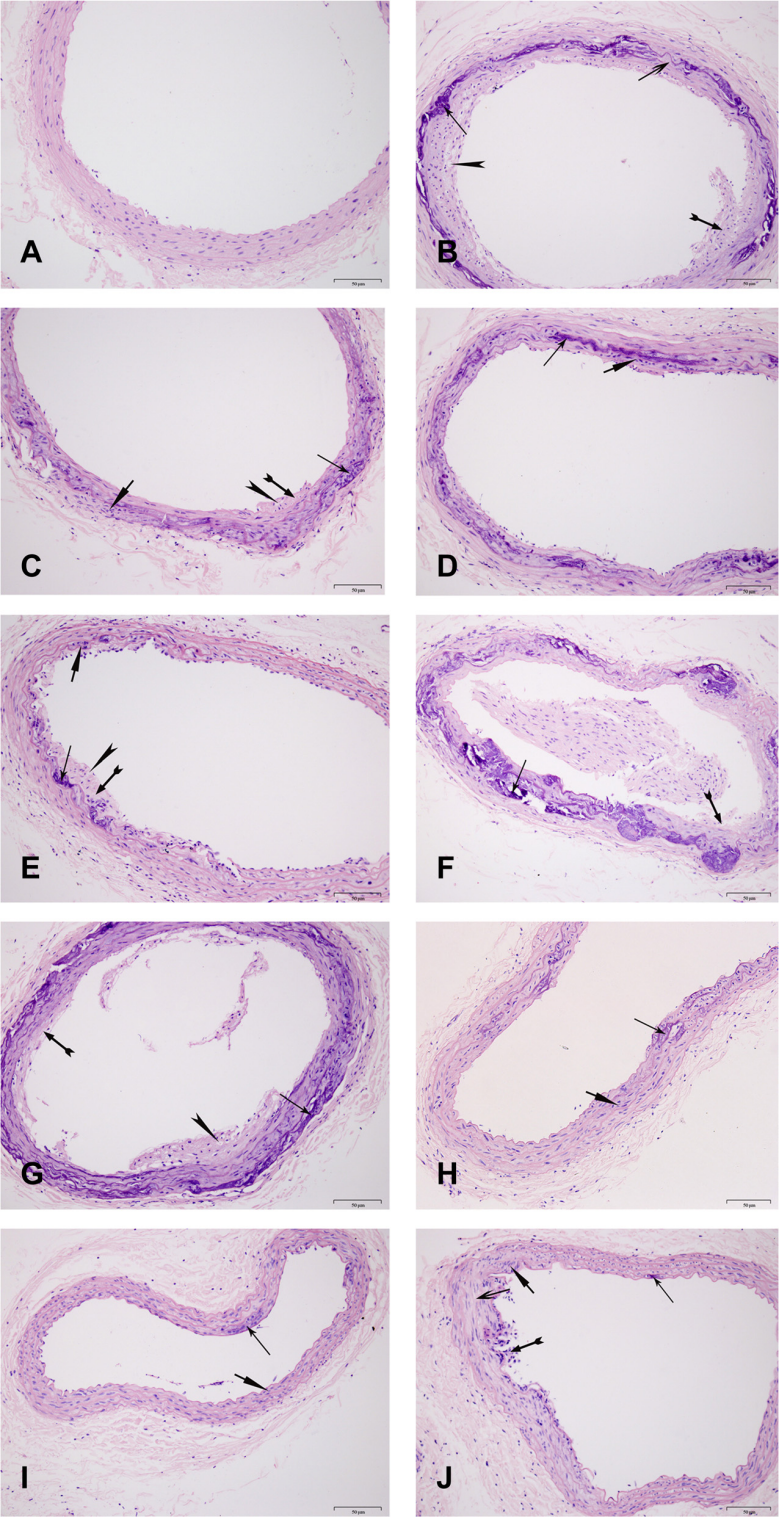
**Effect of different treatment on histopathological changes in LCCA sections by hematoxylin-eosin-stained (magnification, ×200).** : deposit of calcium : deposit of lipid; : intima injury; : smooth muscle cells proliferation; : elastic fiber injury. **A)** The sham group sample: The structure of artery including intimal layer, medial layer and adventitia was clear; **B)** The model group sample (0 g/kg YXC × 0 h swimming): The arterial wall thickening was about 1/4 of A. Lipid in the intima increasing, deposit of calcium and smooth muscle hyperplasia of media were seen. And elastic fiber was damaged; **C)** 0 g/kg YXC × 0.5 h swimming group sample: The focal artery wall thickening, lipid and smooth muscle were seen; **D)** 0 g/kg YXC × 1 h swimming group sample: Obvious artery wall thickening was not seen, and smooth muscle of media increased slightly; **E)** 1 g/kg YXC × 0 h swimming group sample: Focal artery wall thickening, lipid deposit, focal deposit of calcium and slight smooth mucle cells were observed and less than group B; **F)** 1 g/kg YXC × 0.5 h swimming group sample and **G)** 1 g/kg YXC × 1 h swimming group sample: There were not obvious changes compared with model group; **H)** 2 g/kg YXC × 0 h swimming group sample: Smooth muscle of media increased slightly and focal deposit of calcium was seen; **I)** 2 g/kg YXC × 0.5 h swimming group sample: Smooth muscle of media increased slightly and focal deposit of calcium was seen in media.; and **J)** 2 g/kg YXC× 1 h swimming group sample: The focal endothelial cell injury and deposit of calcium were found. The abnormal changes can been seen in part of elastic fiber. The initial state of atherosclerosis was found in the section **(B)** of the model rats. The pathological changes were suppressed to some degree by the combination of 2 g/kg YXC and swimming **(H-J),** swimming alone **(C, D)** or 1 g/kg YXC alone **(E)**.

The sham-operated group showed no histopathological changes (Figure 3A). In the model group (Figure 3B), the vessel wall appeared to be thicker, and lipids in the intima were observed. The deposit of calcium, smooth muscle hyperplasia of media, and elastic fiber injury were also found. Based on the standard histological classification of AS lesions from the American Heart Association [37], the animal model was in the initial stage of AS. Histopathological findings from swimming groups (Figure 3C,D) and YXC (Figure 3E,H) showed less deposit of calcium and light intima injury. Only just a little lipid was seen in treatment group 0.5 h swimming (Figure 3C) and group 1 g/kg YXC (Figure 3E). The changes of interaction between 1 g/kg YXC with 0.5 h swimming (Figure 3F) and with 1 h swimming (Figure 3G) in pathological was not found comparing with model group. However a bit smooth muscle cell proliferation and deposit of calcium were detected in the combined groups of 2 g/kg YXC with 0.5 h swimming (Figure 3I) and with 1 h swimming (Figure 3J). The other pathlogical changes were not so obvious (see Figure 3). The above described observation exhibited that there existed interactive effects between 2 g/kg YXC and swimming in attenuating pathology in the initial stage of AS.

### Assessment of vascular construction remodeling

Compared to the sham-operated group, the wall thickness in the model group was significantly greater (28.65 ± 5.66 μm vs. 19.40 ± 4.21 μm) (p < 0.05), the circumference was significantly smaller (678 ± 73.86 μm vs. 758 ± 57.26 μm) (p < 0.05).

The significant interactive effects between YXC and swimming were only obvious in decreasing the wall thickness (F = 2.350, p = 0.047) (see Table 4).

**Table 4:** **Structural remodeling measurement of different treatment (mean ± SD) (n = 8, each)**

**YXC (g/kg)**	**Swimming (h)**	**Wall thickness (um)**		**Circumference (um)**	
0	0	28.65 ± 5.66		678.91 ± 73.86	
0	0.5	22.91 ± 4.01		744.99 ± 64.06	
0	1	21.33 ± 2.55		731.37 ± 59.84	
1	0	25.46 ± 5.75		706.91 ± 86.70	
1	0.5	22.88 ± 4.14		680.58 ± 50.25	
1	1	22.61 ± 5.82		714.39 ± 65.46	
2	0	21.46 ± 2.81		719.61 ± 58.98	
2	0.5	22.67 ± 2.66		699.70 ± 63.79	
2	1	24.28 ± 4.69		736.37 ± 79.41	
**Two-way ANOVA**		***F***	***P***	***F***	***P***
YXC		0.577	0.565	1.195	0.310
Swimming		2.088	0.133	0.291	0.748
YXC × Swimming		2.350	0.047	0.171	0.952

All data are expressed as the mean ± SD. The GLM procedure was applied for a two-way ANOVA to test for synergism effect. Comparisons between each pair of factor levels were analyzed using a Bonferon post hoc test. A value of P < 0.05 was considered statistically significant.

### Assessment of vascular functional remodeling

**Figure 4:**
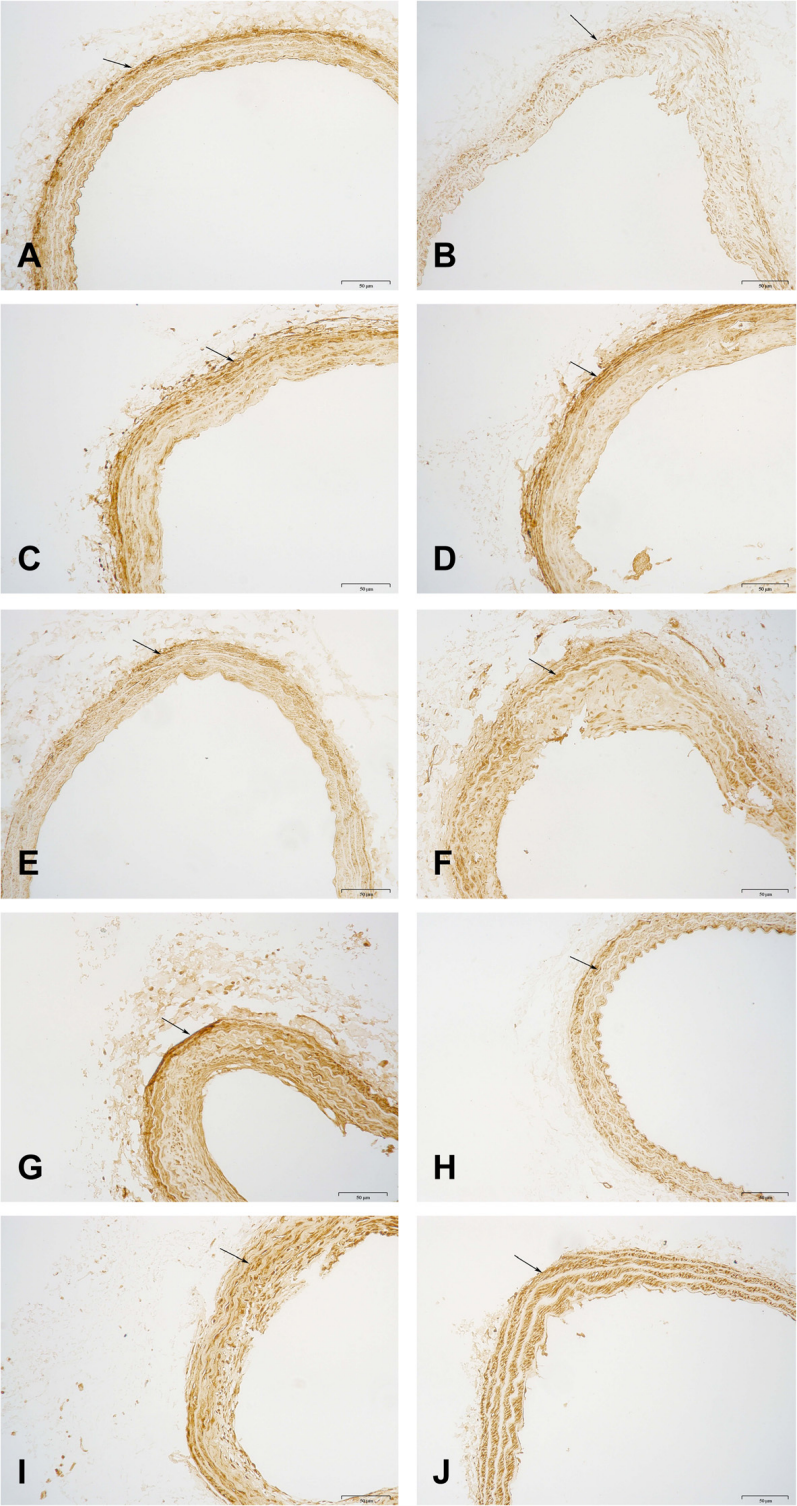
**Effect of different treatment on SM22α protein expression in LCCA sections by immunohistochemistrial staining (magnification, ×200). A)** The sham group sample; **B)** The model group sample; **C)** 0.5 h swimming group sample; **D)** 1 h swimming group sample; **E)** 1 g/kg YXC group sample; **F)** 1 g/kg YXC and 0.5 h swimming group sample; **G)** 1 g/kg YXC and 1 h swimming group sample; **H)** 2 g/kg YXC group sample; **I)** 2 g/kg YXC and 0.5 h swimming group sample; and **J)** 2 g/kg YXC and 1 h swimming group sample.

The result of immunohistochemistry staining showed that compared with the sham group, there was minimal vascular staining (light yellow), and the relative levels of SM22α were significantly decreased (5.27 ± 0.94 vs. 3.20 ± 0.87; p < 0.05) in the model group. When different treatments were applied, SM22α was up regulated to different degrees, and the staining was darker (see Figure 4).

**Figure 5:**
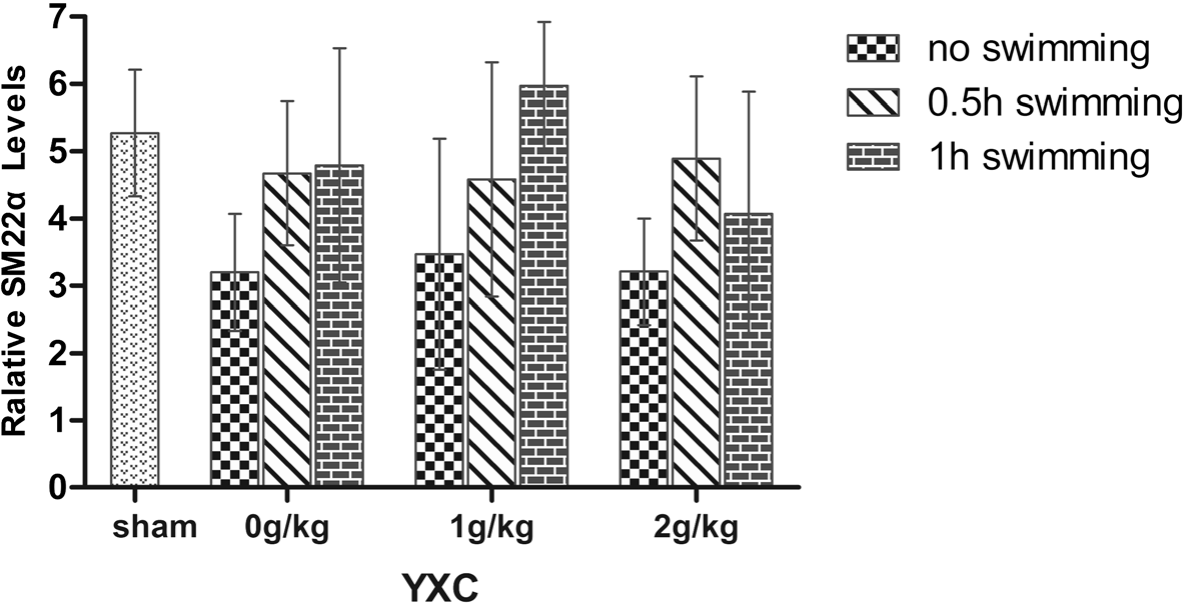
**Relative SM22α levels in different groups.** The GLM procedure was applied for a two-way ANOVA to test for synergism effect. Comparisons between each pair of factor levels were analyzed using a Bonferon post hoc test. A value of *P* < 0.05 was considered statistically significant.

The main effect of swimming on SM22α protein level was significant (F = 7.728, p = 0.001). YXC groups had no influence on the relative levels of SM22α. The combined treatment with YXC and swimming increased relative levels of SM22α to different degrees, revealing the interactive effects between YXC and swimming in promoting vascular functional remodelling (see Figure 5).

## Discussion

Despite changes in lifestyle and the application of new pharmacologic approach, cardiovascular diseases continuously are the principal cause of death. The lesions of atherosclerosis represent a series of highly specific cellular and molecular responses that can be described not only a lipid disorder, but also a highly characteristic process of endothelial dysfunctions [22]. In fact, shear stress-related events are closely related to the initiation and development of AS [38]. Recent investigations show that the maintenance of physiological laminar shear stress is crucial for the regulation of blood flow, the inhibition of proliferation and inflammation of EC, the abnormal shear stress caused by disturbed or oscillatory flows near arterial bifurcations, branch ostia and curvatures play a key role in the procession of AS. Cheng et al. [39] demonstrated that lowered shear stress induces atherosclerotic plaque, whereas increased shear stress protects against atherosclerosis. Thus, whether the regulation of shear stress will become a new therapeutic target for treating AS is worth to be seriously considered. Fortunately, exercise, as a whole body periodic acceleration and enhanced external conterpulsation, is widely recognized as non-invasive methods to intervene some cardiovascular diseases by elevating local or systemic shear stress. The findings from Yun You [28] indicated that the combined therapy of oral Chinese medicine with swimming can inhibit inflammatory factors, improve hemorheological parameters and lipoproteins in a rat AS model induced by combination of low shear stress resulted from partial ligation of common carotid artery with afterwards feeding of a hyperlipotic diet. It may provide the insight into that-the modulation of blood shear stress by exercise may-perform as a “multi-targeted drug” against the some pathological proneness like hypertension and AS.

In the present study, we employed an early AS model in rats which was induced by a high-fat diet, partial ligation of LCCA and vitamin D3 injection to investigate the joint effect of combined Chinese medicine and exercise training. The results showed that the combined treatment of swimming and YXC significantly attenuated the early pathological changes and improved hemorheological properties, the alleviation of lipoprotein retention and endothelial injury by the combined intervention was beneficial for the inhibition of AS.

Hemorheology play a critical role in initiation and development of AS. The main factors of impacting hemorheology include blood viscosity, plasma viscosity, FIB, HCT and so on. The changes of these factors directly influence the blood fluidity, viscosity and coagulation and have become a risk event for AS. In the study, the joint effects of YXC and swimming indicated a significant decrease in plasma viscosity ((F = 3.276, p = 0.016), blood viscosity at 10 s^−1^ (F = 3.192, p = 0.018) and 200 s^−1^ (F = 5.263, p = 0.001) FIB (F = 4.052, p = 0.005) and HCT (F = 2.554, p = 0.047). Specially, the combination of 2 g/kg YXC and swimming exhibited more obvious effect. This finding suggested that the interaction between 2 g/kg YXC and swimming can be contributed to the prevention of AS by improving hemorheological parameters.

Many studies have shown that abnormal lipid metabolism is an important factor in the occurrence and development of AS [40,41]. The increase of LDL-C accompanied by a decrease in HDL-C is considered to be a dangerous factor for evolution of AS [42]. In the present study, the concentrations of HDL-C, LDL-C, TG and TC were detected. Results indicated that there were significant interactive effects between YXC and swimming in LDL-C (F = 3.950, p = 0.028) and TG (F = 5.480, p = 0.001), which suggested that the combination of YXC and swimming played a significant role in improving blood lipids, and 2 g/kg YXC and 1 h of swimming was the optimum combination,, through the effect of YXC was slightly stronger than swimming in improving the blood lipid profile.

Numerous pathophysiologic observations in humans and animals led to the accormulation of the response-to-injury hypothesis of atherosclerosis, which initially proposed that endothelial dysfunction was the first step in atherosclerosis. And the levels of NO, ET, 6-keto-PGF1α and TXB_2_ are closely associated with endothelial dysfunction, which has been suggested to be early markers of AS. The decrease of nitric oxide (NO), the increase of endothelin (ET) and alteration in ratio of 6-keto-PGF1α, and TXB_2_ are the important characters of endothelial dysfunction, which can promote atherosclerotic lesion formation [43]. In the present study, there existed a significant interaction between YXC and swimming in increasing NO and 6-keto-PGF1α and decreasing ET and TXB_2_. The combined treatment of 2 g/kg YXC with 0.5 h swimming and 2 g/kg YXC × 1 h swimming) demonstrated obvious effects in increasing NO and 6-keto-PGF1α and decreasing ET and TXB_2_, swimming alone had significant effect in decreasing TXB_2_ (F = 5.479, p = 0.001). The demonstration suggested that the combination of YXC and swimming could retard AS development by protecting the vascular endothelium.

Evidences indicate that hypertrophy is the main type of vascular remodeling in patients with AS [44]. The remodeling is characterized by an increase of intima-media membrane area within the blood vessel wall, and a narrow of blood vessels in diameter, which was due to vascular smooth muscle cell proliferation. In the present study, thickness of the blood vessel walls in the model group was obviously greater than that in sham group, and the circumference was clearly reduced. This result was consistent with previously reported results [45]. The combination of swimming and YXC also reduced the wall thickness significantly (F = 2.350, p = 0.047). This result suggests that changes of the wall thickness were contributed to inhibition of vascular remodeling by combination of YXC and swimming.

Phenotypic modulation of vascular smooth muscle cells (VSMCs) plays a key role in vascular remodeling diseases, such as AS, hypertension and restenosis, and is a process common to all of the pathophysiological changes. Recently, SM22α has been considered a marker of contractile smooth muscle cells (SMCs) and vascular remodeling, which is often used for the screening of anti-vascular remodeling drug. Especially, SM22α modulates vascular smooth muscle cell phenotype during development of atherogenesis. In our study, SM22α was examined in the LCCA via immunohistochemistry. We found that the expression of SM22α decreased significantly in the model group, which was consistent with the results of Feil [46] and Wamhoff BR [47]. After treatments with YXC and swimming, the expression of SM22α increased to different degrees, and 0 h swimming, 1 h swimming and 2 h swimming indicated a more obvious influence. We speculated that swimming may inhibit vascular remodeling by intervening in the phenotypic transformation of mature SMCs and further inhibit the development of atherosclerotic plaques.

Endothelial cells and smooth muscle cells are the main units of the composition and function of vessels walls. Vascular remodeling often occurs after endothelial injury and leads to smooth muscle cell proliferation. When the changed hemodynamic environment occurs, endothelial cells can sense and transmit the stimulus, playing an important role, in conjunction with smooth muscle cells, in the process of vascular remodeling through releasing a series of substances [48]. L Wang et al. [49] found that after co-culturing endothelial cells (ECs) with smooth muscle cells treated by low shear stress (LSS), ECs could affect the proliferation and phenotypic transformation of smooth muscle cells via IGF-1R, Akt phosphorylation and Sirt2 expression. The present study showed that swimming could markedly enhance the expression of SM22α, a protein associated with the phenotypic transformation of smooth muscle cells. According to L Wang’s conclusion, the present study inferred that swimming might suppress the phenotypic transformation of smooth muscle cells by affecting endothelial cells and inhibiting the process of vascular remodeling.

There is no doubt that the therapeutic interventions in prevention and treatment of AS has gained popularity. However, an important consideration is whether these interventions effectively stop the procession of AS? The application of Chinese medicines has a long history in China, and the potential benefits in prevention and treatment of cardiovascular diseases has been widely accepted [50,51]. Currently, researchers found that disturbed shear stress influences the site selectivity of atherosclerotic plaque formation and its associated vessel wall remodeling, whereas high shear stress might be like a drug with biological reoponce and play a helpful role in treating AS. Furthermore, biomechano-pharmacology, a new borderline discipline [28] is forming between biomechanics and pharmacology. The discipline will probably consist of both the pharmacological intervention of signals induced by biomechanical factors and the biomechanical influence on pharmacokinetics and pharmacodynamics. In fact, the impact of exercise on health has been recognized since the ancient time. To date, it has become increasingly clear that exercise, as a moderate-intensity physical activity, can prevent cardiovascular disease because it can elevate systematic blood circulation to promote blood shear stress. Hence, the combined biological effects derived from drugs and biomechanical forces may become a prospect for preventing and treating AS.

In this study, the combination of YXC and swimming was used to investigate the joint effect of the combined therapy in preventing and treating AS in rats. Our results demonstrated that this combination explored significant interactive effects on the improvement of hemorheological disorders, the pathological changes in the early atherosclerosis and blood lipid profile. Furthermore, the ameliorated vascular endothelial function and the increased vascular remodeling marker SM22α can be contributed to one of mechanisms of the synergistic reaction. The demonstration reveals that the intervention of atherosclerosis by synergistic effects derived from exercise and a traditional Chinese medicine should be a promising perspective. Therefore, to benefit from the new discipline, going with a biomechanopharmacologically tailored exercise should be advocated, through the coming into clinical use of biomechanopharmacology still needs more time, greater efforts and popular awareness from doctors and patients.

## Conclusions

In conclusion, this study indicates that the combination of YXC and swimming may prevent atherosclerosis through a synergistic effects t between YXC (a medicinal factor) and swimming (a physical exercise ) in improving blood circulation, hemorheological parameters, blood lipids levels and the vascular endothelium in rats. The vascular remodeling may contributed to the prevention effects on AS by up-regulating SM22α.

## Abbreviations

6-keto-PGF1α: 6-keto- prostaglandin F_1α_
AS: Atherosclerosis
ET: Endothelin
FIB: Fibrinogen
HCT: Haematocrit
HDL-C: High-density lipoprotein-cholesterol
HE: Hematoxylin and eosin stain
LCCA: Left common carotid artery
SM22α: Smooth muscle 22 alpha
TC: Total cholesterol
TG: Triglyceride
TT: Thrombin time
TXB_2_: Thromboxane B_2_
YXC: Yindan xinnao capsule
